# Redox/pH-responsive hollow manganese dioxide nanoparticles for thyroid cancer treatment

**DOI:** 10.3389/fchem.2023.1249472

**Published:** 2023-09-15

**Authors:** Jinren Liu, Changzhi Guo, Chunxiang Li, Qiushi Jia, Zhengrong Xie, Ziyue Wang, Hongda Tian, Zhongyuan Li, Liguo Hao

**Affiliations:** ^1^ Department of Molecular Imaging, School of Medical Technology, Qiqihar Medical University, Qiqihar, China; ^2^ Department of Molecular Imaging, The First Affiliated Hospital of Qiqihar Medical University, Qiqihar, China

**Keywords:** hollow manganese dioxide, cisplatin, nano drug delivery system, magnetic resonance imaging, enhanced permeability and retention effect

## Abstract

The nano drug delivery system MnO_2_/CDDP@PDA-Cy5.5 was synthesized in this study to increase the efficacy of Cisplatin (CDDP) on thyroid cancer and alleviate the damage to normal tissue, with the aim of enhancing the anti-cancer efficacy, increasing the drug load, optimizing the control of drug release, and alleviating the systemic toxicity arising from drug off-target. On that basis, high efficacy and low toxicity win-win can be obtained. In this study, hollow manganese dioxide nanoparticles (MnO_2_ NPs) were prepared based on the template method. CDDP was loaded into the hollow cavity and then modified with polydopamine (PDA) and Cy5.5, with the aim of obtaining the nano-drug loading system MnO_2_/CDDP@PDA-Cy5.5 NPs. The NPs precisely delivered drugs by intelligently responding to the tumor microenvironment (TME). As indicated by the release curves, the NPs release CDDP rapidly by inducing the decomposition of PDA and MnO_2_ under acidic or redox conditions, and Magnetic resonance imaging (MRI) contrast agent Mn^2+^ was generated. The results of the *in vivo* MRI studies suggested that T_1_ contrast at the tumor site was notably enhanced under the Enhanced permeability and retention (EPR) effect. After the intravenous administration, the effective tumor accumulation exhibited by the NPs was confirmed by magnetic resonance imaging as a function of time. Compared with free CDDP, the *in vivo* therapeutic effect was remarkably increased. As indicated by the above-described results, MnO_2_/CDDP@PDA-Cy5.5 NPs is a drug delivery system exhibiting diagnostic and therapeutic functions.

## 1 Introduction

Thyroid cancer (TC) refers to a malignant tumor originating from the follicular epithelium or parafollicular epithelial cells of the thyroid gland. It has been confirmed as the most common endocrine malignancy worldwide (Wiltshire et al., 2016; Zhang et al., 2022). This cancer is the fifth most common cancer among adult women, and the incidence rate of women after 50 is three times that of men (Hassanipour et al., 2023).

The existing treatment method in terms of thyroid cancer comprises surgical removal of the thyroid gland or radiotherapy, or a combination of surgery and radiotherapy (Li et al., 2017). Chemotherapy has not served as a significantly common treatment option for thyroid cancer. However, existing research has suggested that chemotherapy can be induced if cancer in the thyroid is detected at an early stage (Gao et al., 2015).

CDDP refers to a powerful anticancer drug that has been extensively employed to treat ovarian cancer (Cheng et al., 2011; Shen et al., 2017), thyroid cancer (Gao et al., 2015), breast cancer (Maedeh et al., 2016), lung cancer (Song et al., 2012; He et al., 2015), as well as nasopharyngeal cancer (Quan et al., 2018). CDDP has been reported as a metal complex of platinum, acting similar to alkylating agent, with the main target of DNA. CDDP binds to DNA, such that DNA cross-linking is generated, DDP-DNA complex is formed, DNA replication can be interfered, or nuclear and cytoplasmic proteins are bound (Zhang et al., 2014). Despite a powerful anticancer effect of CDDP, its wide application in clinical practice has been severely hindered due to side effects (e.g., renal toxicity and neurotoxicity) (Siddik, 2003; Barabas et al., 2007). To alleviate toxicity, cisplatin derivatives (e.g., carboplatin and oxaliplatin) have been developed. Despite their low renal toxicity, they still exhibit certain myelotoxicity. Only a small amount of the drug can reach the tumor site after intravenous injection of CDDP, and most of the rest is absorbed by normal cells of the body, such that the body is subjected to serious toxic side effects. Accordingly, recent studies are mainly focused on cisplatin targeted drug delivery systems, including polymer (Shi et al., 2015), liposome (Guo et al., 2013), inorganic (Lv et al., 2016) and other solid particles (Hwang et al., 2017), which can transport cisplatin to tumor sites through active or passive targeting, so that cisplatin can be sustained or triggered to release in tumor. The solubility of platinum is improved, its half-life *in vivo* is prolonged, its distribution in tumor sites is increased, and the off-target distribution of platinum is reduced, thus reducing the toxic and side effects of cisplatin on the body to some extent (Oberoi et al., 2013; Farooq et al., 2019).

Inorganic biodegradable materials have attracted rising attention over the past few years for their unique properties and diverse forms. To be specific, manganese dioxide nanomaterials, a unique TME response nanomaterial for cancer therapy, have aroused extensive attention for their high biocompatibility, stable structure, as well as easy-to-modify surface. It has been investigated in terms of drug delivery and controlled release (Ning et al., 2022). The MnO_2_ nano-system is capable of reacting with reduced glutathione (GSH) and H^+^ present in TME to generate paramagnetic Mn^2+^, such that the contrast of T_1_ magnetic resonance imaging (MRI) can be significantly improved (Xu et al., 2022; Brito et al., 2023). It can be employed for tumor-specific imaging and development of multifunctional drug carrier systems (Hao et al., 2016a; Yang et al., 2017; Xu et al., 2021). Besides, unlike other non-biodegradable inorganic nanomaterials, MnO_2_ can be broken down into the harmless water-soluble Mn^2+^ that can be rapidly eliminated by renal metabolism without long-term *in vivo* toxicity (Chen et al., 2016; Zhu et al., 2016; Zhou et al., 2021). Moreover, MnO_2_ nanoparticles exhibiting similar functions to peroxidase are capable of catalyzing the decomposition of considerable H_2_O_2_ in tumors into water and oxygen, such that tumor hypoxia can be relieved, and photodynamic therapy can be enhanced (Chen et al., 2016; Deng et al., 2022; Pi et al., 2023). In addition, MnO_2_ can rapidly degrade and release loaded drugs for their response to TME. In spite of those appealing features of MnO_2_ nanostructures, some currently developed MnO_2_ nanostructures are still lack of well-defined morphology, such as irregular nanosheets or uneven nanoparticles, which are not desirable for optimizing drug loading and controlling drug release. In comparison to those structures, hollow nanostructures with meso-porous shells (e.g., hollow mesoporous silica) and large cavities have been demonstrated to be excellent drug loading/delivery systems to load high quantities of therapeutic agents, whose release may be precisely controlled by tuning the shell structures or coatings (Chen et al., 2014; Li and Shi, 2014; Cheng et al., 2020).

Previous studies have shown that polydopamine is a novel polymer inspired by mussels, which has natural advantages such as good biocompatibility, adhesion and multiple drug response release (Liu et al., 2013; Zheng et al., 2016). Under slightly alkaline conditions, an adhesive film can be formed on the surfaces of various materials regardless of the surface shape. Therefore, in the nanoparticles coated with PDA, PDA can be used as the gate of drug release, which can reduce the premature release of drugs in blood circulation. When nanoparticles enter tumor cells, under acidic conditions, PDA membrane depolymerizes into shorter polypeptide chains, amino acids or even smaller molecules, and at the same time releases encapsulated drugs (Lee et al., 2007). Therefore, PDA is an ideal delivery carrier to realize the intelligent release of drugs in tumor cells.

A multifunctional nanoparticle was reasonably designed in this study for the first time. The chemotherapy-drug CDDP was encapsulated by MnO_2_ and then coated with Cy5.5-functionalized PDA to generate MnO_2_/CDDP@PDA-Cy5.5 NPs. The nanoparticles exhibited tumor targeting and pH responsiveness and the capability of effectively killing tumors by timely releasing cisplatin. The NPs were well shielded against the early release of CDDP in the circulation since the PDA shell was stable at pH 7.4. The PDA shell was broken when NPs entered the tumor location (pH 6.8) under the EPR effect, such that CDDP was released into the acidic environment. Subsequently, the exposed MnO_2_ nuclei in tumor cells reacted with excessive H^+^ to liberate Mn^2+^, and the ensuing Mn^2+^ degradation exhibited noticeable MRI characteristics. Thus, the developed nano drug delivery system with integrated diagnosis and treatment can take on critical significance in improving the treatment plan of thyroid cancer and increasing the cure rate of thyroid cancer.

## 2 Materials and methods

### 2.1 Reagents

Tetraethyl orthosilicate (TEOS), dimethyl sulfoxide (DMSO), CDDP, Dopamine hydrochloride (DA), ammonium hydroxide solution (NH_3_·H_2_O, ∼28% NH_3_ in water) and Anhydrous ethanol originated from Shanghai Maclin Biochemical Technology Co., LTD. Phosphate buffer solution (PBS) and Trimethylol aminomethane hydrochloric acid (Tris-HCl) were purchased from Beijing Coolaber Technology Co., LTD. Moreover, potassium permanganate (KMnO_4_) and sodium carbonate (Na_2_CO_3_) were offered by Henan Yaoye Chemical Products Co., LTD. Amino Cy5.5 (NH_2_-Cy5.5) was purchased from Solarbio Biotechnology Co., LTD. The Millipore system (Milli-Q, 18.2 MX cm) was used to purify deionized water (H_2_O). All other chemicals were received in good time and were of reagent quality.

### 2.2 Synthesis of MnO_2_ nanoparticles

First, solid silica (sSiO_2_) nanoparticles were synthesized. 120 ml anhydrous ethanol, 20 ml deionized water, and 10 ml ammonia were introduced into a 250 ml round bottomed flask in turn. After sealing, the sample was subjected to magnetic stirring for 15 min, and then 4.5 mL ethyl orthosilicate was slowly introduced into the mixed solution in the stirring. After sealing, the stirring continued for 8 h at ambient temperature to obtain SiO_2_ NPs. The resulting SiO_2_ NPs were washed 3 times with anhydrous ethanol and deionized water, respectively. Subsequently, the NPs were stored in deionized water for the next synthesis SiO_2_@MnO_2_.

600 mg KMnO_4_ dispersed in 20 ml water was added dropwise to the stored SiO_2_ NPs after ultrasonic treatment. Next, the mixture was ultrasonically treated for 0.5 h (37°C, 40 KHZ) and then stirred at ambient temperature overnight. The reaction solution was centrifuged at 11,000 rpm/min, the supernatant was discarded, and the obtained precipitates were washed with anhydrous ethanol and deionized water for three times, respectively. On that basis, SiO_2_@MnO_2_ nanospheres with core-shell structure were generated.

Lastly, core-shell SiO_2_@MnO_2_ nanoparticles were dissolved into Na_2_CO_3_ solution for 12 h at 60°C. Subsequently, the etched dispersion was centrifuged, the supernatant was discarded, and the obtained precipitates were washed with anhydrous ethanol and deionized water for three times in turn to obtain MnO_2_ NPs.

### 2.3 Synthesis of MnO_2_/CDDP@PDA NPs

10 mg MnO_2_ was introduced to 10 mL DMSO containing 10 mg CDDP and stir in the dark for 12 h. Subsequently, the remaining CDDP was centrifuged out of the solution to generate MnO_2_/CDDP NPs. The NPs were dispersed in 10 mL Tris-HCl buffer (pH 8.5, 10 × 10^−3^ M) containing 10 mg DA, and stirred in the dark for 6 h at ambient temperature to obtain MnO_2_/CDDP NPs coated with PDA. The final product MnO_2_/CDDP @PDA NPs was centrifuged and lyophilized for 48 h.

### 2.4 Synthesis of MnO_2_/CDDP@PDA-Cy5.5NPs

10 mg MnO_2_/CDDP@PDA NPs and 1 mg NH_2_-Cy5.5 were fully dispersed in 10 ml Tris-HCl buffer and reacted at ambient temperature for 6 h away from light. The mixture was then centrifuged for 10 min at 11,000 rpm/min and washed 3 times with deionized water to remove the remaining NH_2_-Cy5.5 to obtain MnO_2_/CDDP@PDA- Cy5.5 NPs. The finished product was freeze-dried for 48 h.

### 2.5 Characterization of materials

The identical volume of MnO_2_ and MnO_2_/CDDP@PDA- Cy5.5 NPs solutions with ultra-pure water and normal saline as the solvents were placed in Xilin bottles, respectively. After the samples stood for 24 h, photos were taken, such that the stability can be compared and analyzed. The morphology, structure and elemental composition of the nanoparticles were characterized through transmission electron microscopy (TEM, FEI Tecnai F20) and X-ray energy dispersion spectroscopy (EDS, Oxford X-Max 80 T). The nanoparticle size and zeta potential were characterized using the Marvin Nanoparticle Size Analyzer (Nano-ZS90). The valence states of the elements were detected in nanoparticles through X-ray photoelectron spectroscopy (XPS, The United States-Thermo SCIENTIFIC ESCALAB 250 Xi). A specific surface area and pore size were investigated using the Barrett–Joyner–Halenda (BJH) method and the Brunauer–Emmett–Teller (BET) method (Micromeritics Instrument Corp. ASAP2460). Ultraviolet-visible spectra of nanoparticles were examined using ultraviolet spectrophotometer (UV-1285, Shimadzu).

### 2.6 Determination of drug loading and encapsulation efficiency

The content of CDDP was determined by UV-vis to evaluate the CDDP of MnO_2_ loading. Using the method developed previously (Anirudhan et al., 2022), 5,400 μg CDDP powder was dissolved into 5 ml DMSO solution, and 1,080 μg/mL CDDP solution was prepared. The solutions of 540, 216, 108, 21.6 μg/mL CDDP were diluted with deionized water. The absorbance values of the CDDP solutions with different concentrations were examined under 301 nm ultraviolet light, and the standard curve was generated (Supplementary Figure S1). After CDDP and MnO_2_ reacted fully, the resultant reaction mixture was centrifuged to separate the supernatant from the precipitate. The optical density of CDDP in the supernatant was measured using the ultraviolet visible spectrophotometer. In addition, in order to prove that the cavity in manganese dioxide improves the drug loading, the drug loading of SiO_2_ before etching is also investigated. The drug loading rate (DL%) and encapsulation efficiency (EE%) of CDDP was examined indirectly by measuring the amount of CDDP in the supernatant, as expressed below:

$$EE\;\left(\%\right)=\frac{\text{Total}\;\text{drug}\;\text{addition}-\text{The}\;\text{amount}\;\text{of}\;\text{drug}\;\text{in}\;\text{the}\;\text{supernatant}}{\text{Total}\;\text{drug}\;\text{addition}}\times 100\%$$

$$\text{DL }\left(\%\right)=\frac{\text{Total}\;\text{drug}\;\text{addition}-\text{The}\;\text{amount}\;\text{of}\;\text{drug}\;\text{in}\;\text{the}\;\text{supernatant}}{\text{mass}\;\text{of}\;\text{drugs}\;\text{loaded}\;\text{final}\;\text{carriers}}\times 100\%$$

### 2.7 CDDP and Mn^2+^ release experiment

With different solutions as the release medium, 2 mL of MnO_2_/CDDP@PDA (1 mg/mL) solution was dissolved into the dialysis bag, and the dialysis bag was placed in the above beakers containing an appropriate number of media and placed on the magnetic stirrers (37°C, 100 rpm). At 1, 2, 4, 8, 12, and 24 h, 0.5 mL of the solution outside the dialysis bag was removed, and the corresponding release medium of equal volume was introduced. The Mn^2+^ content was analyzed by inductively coupled plasma atomic emission spectrometry (ICP-OES). The content of CDDP was determined using an ultraviolet spectrophotometer, and the CDDP release rate was calculated in accordance with the established standard curve and reference formula. C_n_ denotes the drug concentration in the release medium at the n time point, V represents the total volume of the release medium, C_k_ expresses the drug concentration in the release medium at the k time point, and V_0_ is the sampling volume.

$$\text{The CDDP release percentage }\left(\%\right)=\frac{\text{Cn}\times V+{\sum_{1}^{n-1}C}_{k}\times V_{0}}{m}\times 100\%$$

### 2.8 MRI and relaxation rate of the NPs dispersion

The concentration of Mn^2+^ was determined as 90.788 mg/L by ICP-OES to determine the relaxation rate of MnO_2_/CDDP@PDA-Cy5.5 and assess the MR imaging capabilities of nanoparticles. Next, MnO_2_/CDDP@PDA-Cy5.5 was dispersed in different conditions. After a 4-h incubation, the MRI study was conducted with a 3.0 T magnetic resonance (MR) device. The T_1_ value of MnO_2_/CDDP@PDA-Cy5.5 under different conditions is determined as a function of concentration. The following acquisition parameters were used in T_1_-weighted image acquisitions: field of view = 90 × 90 mm^2^, matrix = 268 × 153, slice thickness = 2.0 mm, repetition time = 3,000 ms, echo time = 80 ms.

### 2.9 Cell culture

8305C human thyroid cancer cells originated from Wuhan Procell Life Science and Technology Co., Ltd., and cultured in MEM (including NEAA) supplemented with 10% fetal bovine serum (Corning), 10,000 U/mL penicillin and 10 mg/mL streptomycin (Corning) in a humidified environment of 37°C and 5% CO_2_. Nthy-ori3-1 human thyroid normal cells were purchased from Zhaoqing Sea Star Biotechnology Co., LTD., and then cultured in RPMI-1640 supplemented with 10% fetal bovine serum (Corning), 10,000 U/mL penicillin, as well as 10 mg/mL streptomycin (Corning).

### 2.10 Cellar uptake experiment

To examine the cell internalization behaviour of various formulations, 8305C cells were incubated with Cy5.5 and MnO_2_/CDDP@PDA-Cy5.5 nanoparticles and then imaged by laser confocal scanning microscopy (CLSM) (Hao et al., 2016b). 8305C cells with a cell density of 2×10^5^/mL were incubated in an incubator for 24 h Cy5.5 and MnO_2_/CDDP@PDA-Cy5.5 were added and incubated for another 8 h. Next, the cells were gradually washed three times with PBS (pH = 7.4). Hoechst was used to identify the nuclei, which were then incubated without light for 30 min. The cells were then given three PBS rinses before 1 mL of PBS buffer was added for CLSM imaging.

### 2.11 *In vitro* cytotoxicity experiment

To assess the cytotoxicity of MnO_2_@PDA-Cy5.5 NPs, 8305C and Nthy-ori3-1 cells were inoculated in 96-well plates at a density of 2×10^5^/well for 12 h. Next, MnO_2_@PDA-Cy5.5 NPs dispersion in culture medium with different concentrations (Based on the concentration of MnO_2_) was introduced to the wells. 10 mL of CCK-8 reagent was added to the relevant well after 24 h. After a light shake, the plates were put back under usual conditions for cell incubation for 0.5–1 h. The microplate reader found the OD value at 450 nm after 0.5 h. Cell viability was calculated using the following formula:

$$Cell\;viability\left(\%\right)=\frac{{OD}_{sample}-{OD}_{blank}}{{OD}_{control}-{OD}_{blank}}$$

In addition, nanoparticles carrying CDDP were used to evaluate the therapeutic effect. In this process, free CDDP, MnO_2_/CDDP@PDA-Cy5.5 (CDDP concentration 0.625, 1.25, 2.5, 5, 10, 20 μg/mL) were incubated with 8305C cells for 24 h, and cell viability was detected by CCK-8 assay. The methods of determination and data processing were the same as those described in the cytotoxicity tests.

### 2.12 Apoptosis experiments

8305C cells with a density of 2×10^5^/mL were inoculated into a 6-well plate to assess the effect of MnO_2_/CDDP@PDA-Cy5.5 on apoptosis of cancer cells. After 12 h incubation, MnO_2_/CDDP@PDA-Cy5.5, MnO_2_, free CDDP, and PBS (blank group) were introduced to the respective well and incubated for another 48 h. The tests conformed to the procedure described in the apoptosis kit. Apoptosis was characterized by flow cytometry.

### 2.13 Animal experiments

Both BALB/c Nude female (15–18 g, 5–6 weeks) and male SD rats originated from Liaoning Changsheng Biotechnology Co., LTD. All animals were used in investigations following a protocol endorsed by the Qiqihar Medical College’s Experimental Animal Ethics Committee. All experimental nude mice were raised in the SPF environment of Medical College with consistent and suitable alternating light and shade, temperature (22°C ± 1°C) and humidity (50%–60%), during which they were fed and drinking water adequately. 8305C cells at logarithmic growth stage were digested with 0.25% trypsin. The cells were collected after centrifugation and resuspended with normal saline. The cell suspension was prepared at a concentration of 2×10^5^/mL and then subcutaneously injected into the right armpit of naked mice. Drug therapy and imaging studies were conducted on the tumor-bearing mice. Weight and tumor volume were examined every 1 day and detailed records were made.

### 2.14 Fluorescence imaging experiments

The Caliper IVIS Spectrum bioluminescence imaging system was employed for fluorescence imaging of tumor-bearing mice. The anesthetized nude mice were placed in a small animal fluorescence imager, and the internal anesthetic gas release device was directed at the mouth and nose of the nude mice for continuous anesthesia. Fluorescence scanning was performed at 1 h, 2 h, 4 h and 12 h before and after MnO_2_/CDDP@PDA-Cy5.5 injection.

### 2.15 Magnetic resonance imaging experiments

After anesthesia, nude mice were placed on a 3.0 T Philips MRI bed for T_1_ signal scanning. After scanning, MnO_2_/CDDP@PDA-Cy5.5 NPs were injected into nude mice by tail vein at a dose of 5 mg/kg. After injecting NPs for 2 h, 4 h and 8 h, the nude mice were scanned for T_1_ signal again, and the results were recorded and then analyzed. The following acquisition parameters were used in T_1_-weighted image acquisitions: field of view = 90 × 90 mm^2^, matrix = 268 × 153, slice thickness = 2.0 mm, repetition time = 3,000 ms, echo time = 80 ms.

### 2.16 *In vivo* tumor treatments

Treatment is initiated when the tumor has grown to 80–100 mm^3^. Mice were randomly divided into four groups with three mice in the respective group. The four groups comprised the PBS group, the MnO_2_ group, the CDDP group, and the MnO_2_/CDDP@PDA-Cy5.5 group. The dose of MnO_2_/CDDP@PDA-Cy5.5 was 3 mg/kg (based on the measurement of CDDP), and the dose of MnO_2_ was 30 mg/kg. The drug was injected into the tail vein of nude mice, once every other day, for a total of 7 times. Before each administration, the weight and tumor volume of mice were examined with balance and vernier caliper, and the health status of mice was observed. The equation for calculating tumor size is V = 0.5×L×S^2^, where V is the volume of the tumor, and L and S are the longest and shortest diameters of the tumor respectively.

### 2.17 Safety assessment distribution and biological

After treatment, nude mice were killed by cervical dislocation, and tumors and major organs (heart, liver, spleen, lung, and kidney) of the respective group were extracted, fixed with 4% paraformaldehyde solution, embedded in paraffin, staining with hematoxylin and eosin (H&E), and imaging with digital microscope (HAMAMATSU S210 microscope). After that, UTP notch end labeling (TUNEL) was used to dye the tumor tissue in order to better determine the tumor’s level of apoptosis. Tumors and major organs (heart, liver, spleen, lung, and kidney) of the probe group were extracted and dissolved into aqua regia for 48h, and Pt^2+^ content was examined by ICP-AES for biological distribution study (Wang et al., 2019).

Biochemical analysis was conducted on 1.5 mL of blood from SD rats administrated with MnO_2_/CDDP@PDA-Cy5.5NPs, and Saline treated rats were assigned into controls. To obtain serum, blood samples were centrifuged at 4,000 rpm for 10 min at 4 C. Subsequently, alanine aminotransferase (ALT), aspartate aminotransferase (AST), creatinine (Cr), and urea nitrogen (BUN) concentrations were measured in the serum with an automatic biochemical analysis (BS-430, Mindray).

### 2.18 Statistical analyses

Data are presented as mean ± standard deviation (SD). The Student's t-test was used for the statistical analysis. The indicators of significance were #*p* > 0.05, *p < 0.05, **p < 0.01, and ***p < 0.001.

## 3 Results and discussion

### 3.1 Preparation of the core-shell MnO_2_/CDDP@PDA-Cy5.5 NPs

**FIGURE 1:**
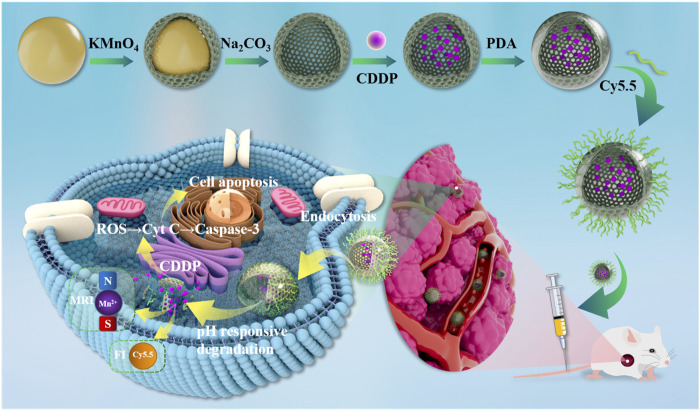
MnO_2_/CDDP@PDA-Cy5.5 NPs synthesis process and the mechanism of diagnosis and treatment.

Figure 1 depicts the synthesis of the MnO_2_/CDDP@PDA-Cy5.5 as well as the mechanism of diagnosis and treatment. In brief, hollow mesoporous MnO_2_ nanoparticles were first prepared by template etching and ultrasonic cavitation. The chemotherapeutic CDDP was then physically encapsulated in a hollow cavity of manganese dioxide nanoparticles to alleviate its inherent toxicity. To precisely control the release of CDDP, PDA shells containing many active groups were formed on MnO_2_ NPs by self-polymerization of dopamine monomers under alkaline conditions to form MnO_2_/CDDP@PDA NPs. In mildly acidic conditions, the PDA shells would depolymerize and release CDDP. Lastly, NH_2-_Cy5.5 is attached to the surface of MnO_2_/CDDP@PDA NPs to form MnO_2_/CDDP@PDA-Cy5.5 NPs through the reaction of amino groups and active esters under weak base conditions.

### 3.2 Characterization of MnO_2_/CDDP@PDA-Cy5.5 NPs

**FIGURE 2:**
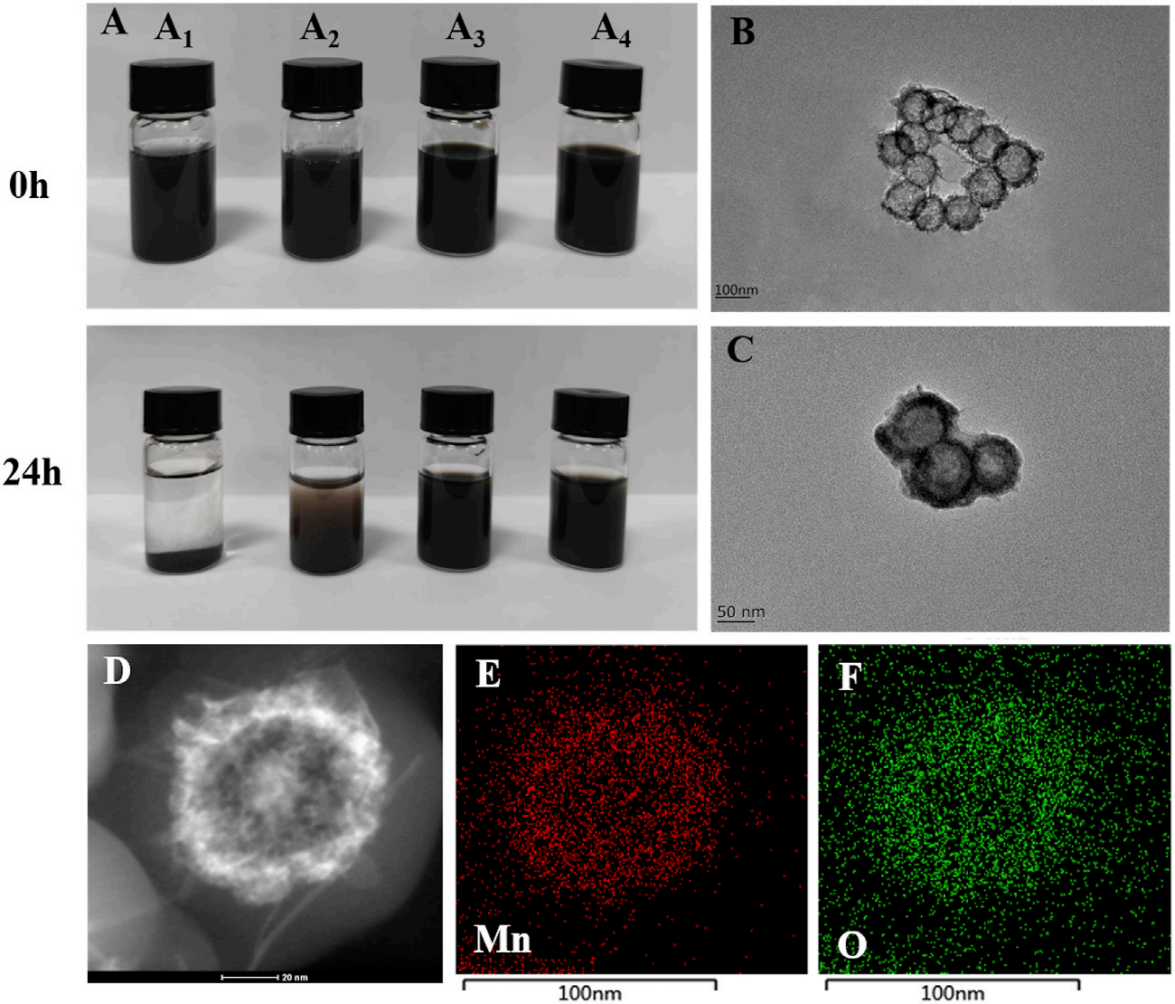
Characterization of MnO_2_ and MnO_2_/CDDP@PDA-Cy5.5 NPs. **(A)** MnO_2_ and MnO_2_/CDDP@PDA-Cy5.5 distributed in pure water and saline at day 0 and day 1, respectively. (A_1_) MnO_2_ in pure water; (A_2_) MnO_2_ in normal saline; (A_3_) MnO_2_/CDDP@PDA-Cy5.5 in pure water (A_4_) MnO_2_/CDDP@PDA-Cy5.5 in normal saline. TEM analysis of **(B)** MnO_2_ NPs and **(C)** MnO_2_/CDDP@PDA-Cy5.5 NPs. **(D–F)** HAADF-STEM image and elemental mapping for MnO_2_.

As can be seen from the comparison before and after Figure 2 (A_1_-A_2_), manganese dioxide nanoparticles are unstable in ultra-pure water and normal saline, and obvious condensation occurs after 24 h of placement. As can be seen from the comparison before and after Figure 2(A_3_-A_4_), the stability of modified MnO_2_ nanoparticles is significantly improved. In addition, the particle size variation of MnO_2_/CDDP@PDA-Cy5.5 NPs in pure water and PBS solution was also studied. As shown in Supplementary Figure S2, the hydrated particle size of MnO_2_/CDDP@PDA-Cy5.5 NPs in pure water and PBS has not changed significantly for seven consecutive days, indicating that it has good stability.

TEM was used to monitor the formation of hollow mesoporous MnO_2_ NPs and core-shell MnO_2_/CDDP@PDA-Cy5.5 NPs. All NPs had strong monodispersity and were generally spherical (Figures 2B, C). Figure 1B’s depiction of the nanospheres’ appearance as having a gray center and an atrous edge indicates that a hollow, mesoporous MnO_2_ structure was successfully synthesized. Additionally, a unique two-layer structure (Figure 2C) indicated that the PDA shell had completely encapsulated the hollow mesoporous MnO_2_ core. Due to the thicker hydration layer, the average particle size of MnO_2_/CDDP@PDA-Cy5.5 NPs generated by TEM was nearly 90 nm, much smaller than that of DLS (Supplementary Figure S4). The TEM size conformed to the theoretical requirements of the EPR effect (Maeda et al., 2013; Wathoni et al., 2020). Furthermore, the element mapping of high-angle annular dark-field scanning (HAADF-STEM) (Figure 2(D-F)) further demonstrated the hollow structure of MnO_2_ and exhibited the capability of distinguishing the manganese and oxygen elements covered in the nanoparticles. Supplementary Figure S3 further quantifies manganese and oxygen, hollow nanoparticles are characterized by higher element density in the edges and lower in the core.

**FIGURE 3:**
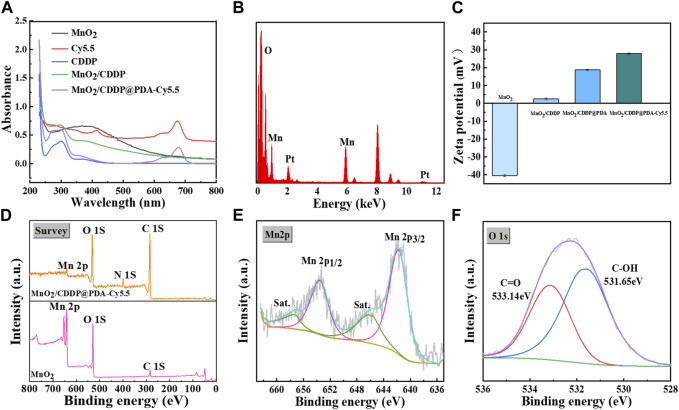
Characterization of MnO_2_ and MnO_2_/CDDP@PDA-Cy5.5 NPs. **(A)** UV–vis spectra of different formulations. **(B)** EDS pattern of MnO_2_/CDDP@PDA-Cy5.5 NPs **(C)** Potential of different nanoparticles. **(D–F)** XPS analysis of as-prepared MnO_2_ NPs and MnO_2_/CDDP@PDA-Cy5.5 NPs, **(D)** Full scan; **(E)** Mn 2p spectra and **(F)** O 1s spectra of MnO_2_/CDDP@PDA-Cy5.5 NPs.

The UV-vis absorption spectra of different preparations were tested, as presented Figure 3A, to verify the successful loading of CDDP into the manganese dioxide cavity and the successful coupling of Cy5.5 with MnO_2_/CDDP@PDA. After CDDP was loaded into MnO_2_, the strong absorption band of MnO_2_/CDDP@PDA-Cy5.5 NPs and MnO_2_/CDDP at 301 nm was evident, representing the characteristic peak of CDDP compared with pure MnO_2_ NPs. MnO_2_/CDDP@PDA-Cy5.5 NPs also displayed an absorption peak at 675 nm, consistent with the absorption wavelength of Cy5.5, suggesting that MnO_2_/CDDP@PDA was successfully coupled with Cy5.5. Furthermore, EDS of MnO_2_/CDDP@PDA-Cy5.5 NPs indicated the presence of platinum elements in Figure 3B, suggesting that CDDP was well loaded into the manganese dioxide cavity, consistent with the change of Zeta potential in the preparation process (Figure 3C).

**FIGURE 4:**
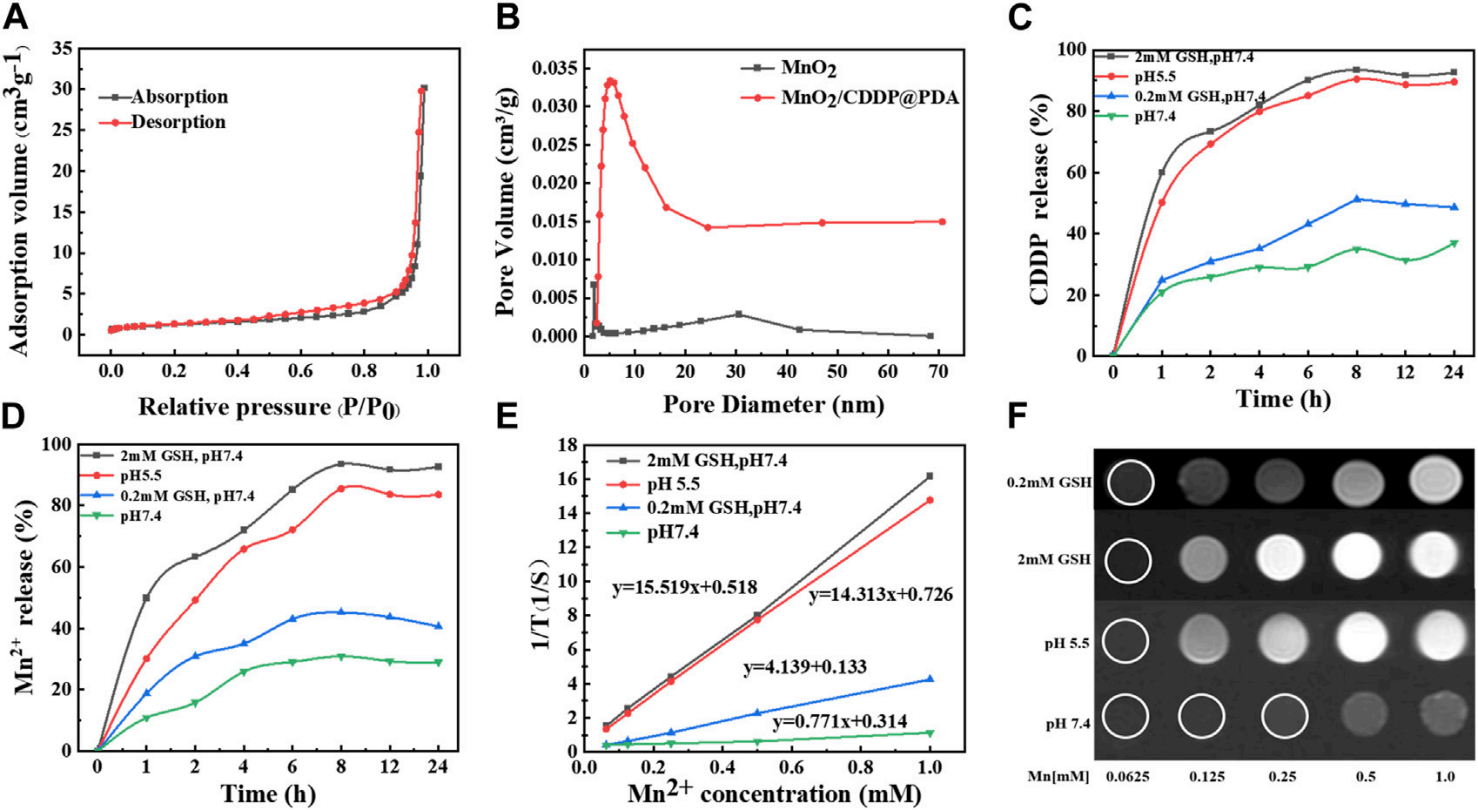
Characterization of MnO_2_ and MnO_2_/CDDP@PDA-Cy5.5 NPs. **(A)** Nitrogen absorption–desorption isotherm and **(B)** pore distribution of MnO_2_ and MnO_2_/CDDP@PDA. **(C)** CDDP and **(D)** Mn^2+^ release from MnO_2_/CDDP@PDA under different conditions. **(E, F)** T_1_-weighted MR images and the transverse relativities (r_1_) of MnO_2_/CDDP@PDA-Cy5.5 dispersion at different conditions.

XPS spectra provide further evidence for the successful formation of the PDA coating (Figure 3D) (Zeng et al., 2020). In other words, the spectrum of MnO_2_ NPs coated with PDA revealed a brand-new N-peak corresponding to the amino group of PDA, suggesting that the preparation of core-shell MnO_2_/CDDP@PDA NPs was successful. According to Figure 2E, the binding energies of the Mn 2p3/2 and 2p1/2 were centered at 642 eV and 653.9 eV, respectively, suggesting that Mn’s primary oxidation state was +4. Figure 2F shows the binding energy of C-OH and C=O centered at 531.65 eV and 533.14 eV, respectively. The nitrogen adsorption–desorption isotherm of MnO_2_ was collected, and the corresponding specific surface area was calculated to be 64.04 m^2^ g^−1^ (Figure 4A). Beyond that, this adsorption–desorption isotherm belongs to the typical V-type isotherm, confirming its rich mesopores. The BJH model was employed to fit the corresponding pore size distribution curve. As indicated by the results, the pore size of MnO_2_ before drug loading was 8.76 nm, and the pore size of MnO_2_/CDDP decreased to 5 nm after drug loading. This variation indicates that cisplatin enters the hollow cavity through the hole (Figure 4B). According to the XRD statistics in Supplementary Figure S5, the distinctive MnO_2_ diffraction peaks at 2θ = 22.4°, 37.1°, 42.6°, 56.4°, and 67.6°, which emerged at (120), (131), (300), (160), and (003), respectively, and corresponded to the MnO_2_ crystal type (JCPDS 14–0644). The above results suggested that the obtained MnO_2_ displayed a cubic crystal structure (Wang et al., 2023).

### 3.3 Amounts of loading drugs, CDDP and Mn^2+^release

In order to increase the loading efficiency of CDDP, the loading efficiency was investigated at different feed ratios. As shown in Supplementary Figure S6, when the ratio of MnO_2_ to CDDP was 1:1, the drug loading rate and encapsulation efficiency reached the maximum, which were 32.27% and 47.65%, respectively, which were 10.82% and 20.34% higher than those before SiO_2_ etching. On that basis, the drug release performance of MnO_2_/CDDP@PDA at different conditions was evaluated. As depicted in Figures 4C, D, Mn^2+^ and CDDP were rapidly released from MnO_2_/CDDP@PDA under 2 mM GSH or acidic conditions (pH = 5.5), but released slowly in neutral PBS. Compared with the slow drug-release profiles of MnO_2_/CDDP@PDA at pH 7.4, the release speeds of both CDDP and Mn^2+^ were found to be faster in weak redox environment. The above-mentioned significant pH and redox-responsive drug release behaviour led to the decreased CDDP release in a neutral environment, prolonged the blood circulation in the body, and facilitated the CDDP release of free pharmaceuticals in times, such that negative side effects on healthy tissues were reduced. Degradation of MnO_2_/CDDP@PDA in acidic solution (pH 5.5) was also observed by TEM (Supplementary Figure S7). We found that the nanoparticles were distributed in sheet form after acid dissolution.

### 3.4 Magnetic resonance imaging and relaxation rate experimental results

As depicted in Figures 4E, F; Supplementary Figure S8A, at pH 7.4, no significant difference was reported in MRI T_1_-weighted imaging signal intensity of MnO_2_/CDDP@PDA-Cy5.5 NPs different concentrations, and the relaxation rate reached 0.771 mM^−1^S^−1^. As revealed by the above-mentioned result, MnO_2_/CDDP@PDA-Cy5.5 NPs did not exhibit any T_1_-weighted imaging ability in neutral environment. Under 2 mM GSH or acidic conditions (pH = 5.5), the signal intensity of T_1_-weighted MnO_2_/CDDP@PDA-Cy5.5 NPs with different concentrations was notably enhanced, and the relaxation rate were 15.519 and 14.313 mM^−1^S^−1^. The possible reason for the above results may be the responsive degradation of MnO_2_/CDDP@PDA-Cy5.5 NPs. Under acidic and redox conditions, MnO_2_/CDDP@PDA-Cy5.5 NPs is reduced to Mn^2+^, which has MRI T_1_-weighted imaging capability.

To evaluate whether the nanoparticles would still display redox responsive MR switchable properties after addition of biologically relevant concentrations of reducing agents, MnO_2_/CDDP@PDA-Cy5.5 (Mn = 1 mM) NPs were treated with different concentrations of GSH at pH = 7.4 and imaged under 3.0 T MR. It can be clearly seen from Supplementary Figure S8B that at this pH, when GSH concentration is between 20 and 200 µM, imaging switching will occur.

### 3.5 Cellar uptake experimental results

**FIGURE 5:**
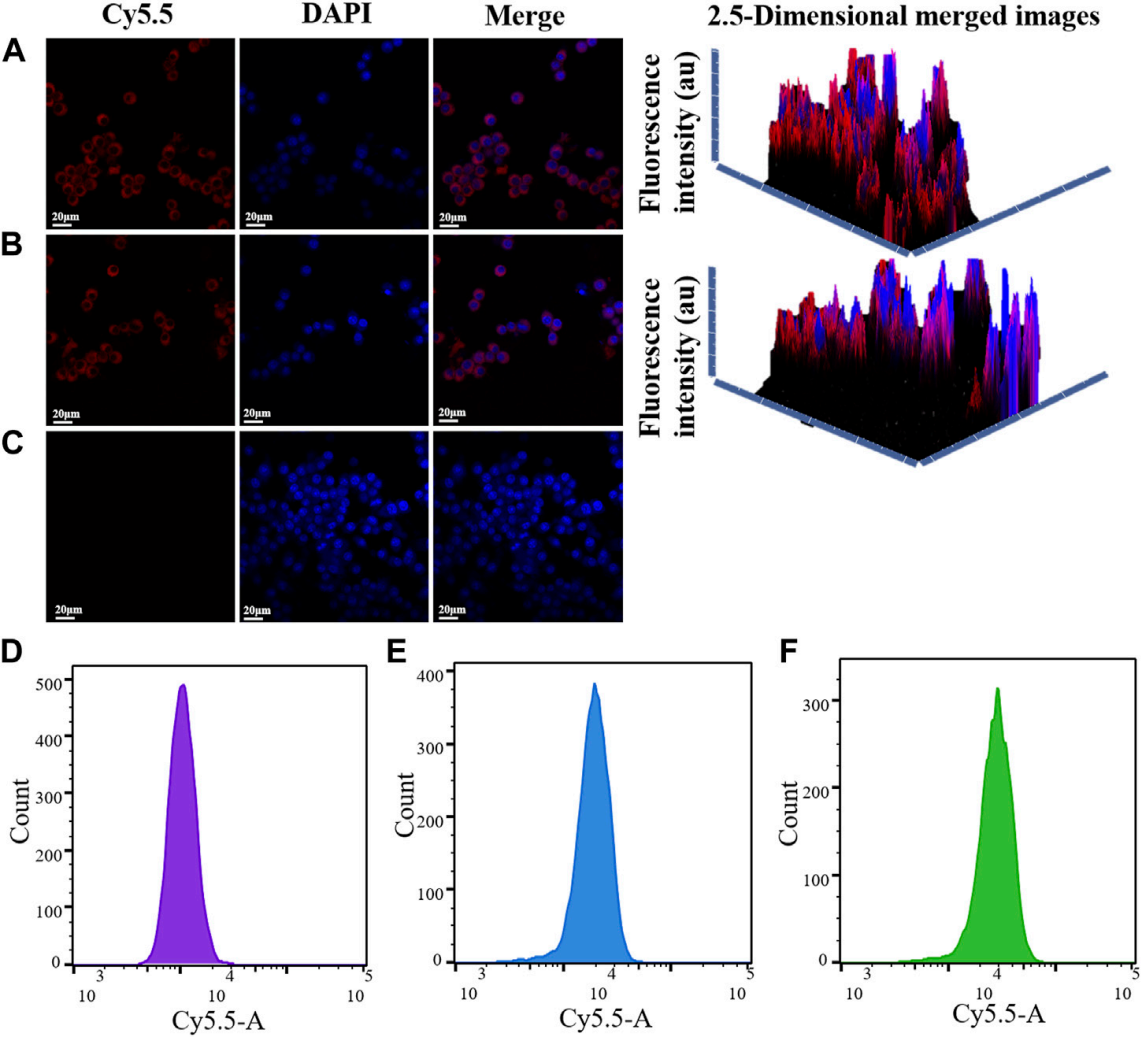
CLSM images of 8305C cells incubated with MnO_2_/CDDP@PDA-Cy5.5 **(A)**, free Cy5.5 **(B)** and PBS**(C)** for 4 h at 37 C. DAPI and Cy5.5 are represented by the colors blue and red, respectively. The red fluorescence of 8305C cells in PBS group **(D)**, Cy5.5 group **(E)** and MnO_2_/CDDP@PDA-Cy5.5 group **(F)**was detected by flow cytometry.

The uptake of MnO_2_/CDDP@PDA-Cy5.5 and its intracellular behaviour were observed through CLSM in depth. As depicted in Figure 5, the cell nuclei stained by DAPI are represented by blue, while Cy5.5 and MnO_2_/CDDP@PDA-Cy5.5 are all represented by red. Notably, MnO_2_/CDDP@PDA-Cy5.5 exhibited greater fluorescence intensity in the cytoplasm. Fluorescence pictures of 8305C cells treated with either MnO_2_/CDDP@PDA-Cy5.5 or free Cy 5.5 were reconstructed in 2.5 dimensions by ZEN 2012 software by utilizing the “optical sectioning” capability of CLSM. Compared with free Cy5.5, MnO_2_/CDDP@PDA-Cy5.5 entered the cell more, especially with stronger red and blue fluorescence, as evidenced by the presence of a fluorescence signal along the z-axis. In order to further study the uptake of MnO_2/_ CDDP@PDA-Cy5.5 by 8305C cells, we carried out fluorescence quantification by flow cytometry. As shown in Figure 7(D-F), The mean fluorescence intensity of MnO_2_/CDDP@PDA-Cy5.5 incubation group was significantly higher than that of Cy5.5 group. These results suggested that the uptake ability of 8305C cells to MnO_2_/CDDP@PDA-Cy5.5 is better than Cy5.5.

### 3.6 Cytotoxicity experimental results

**FIGURE 6:**
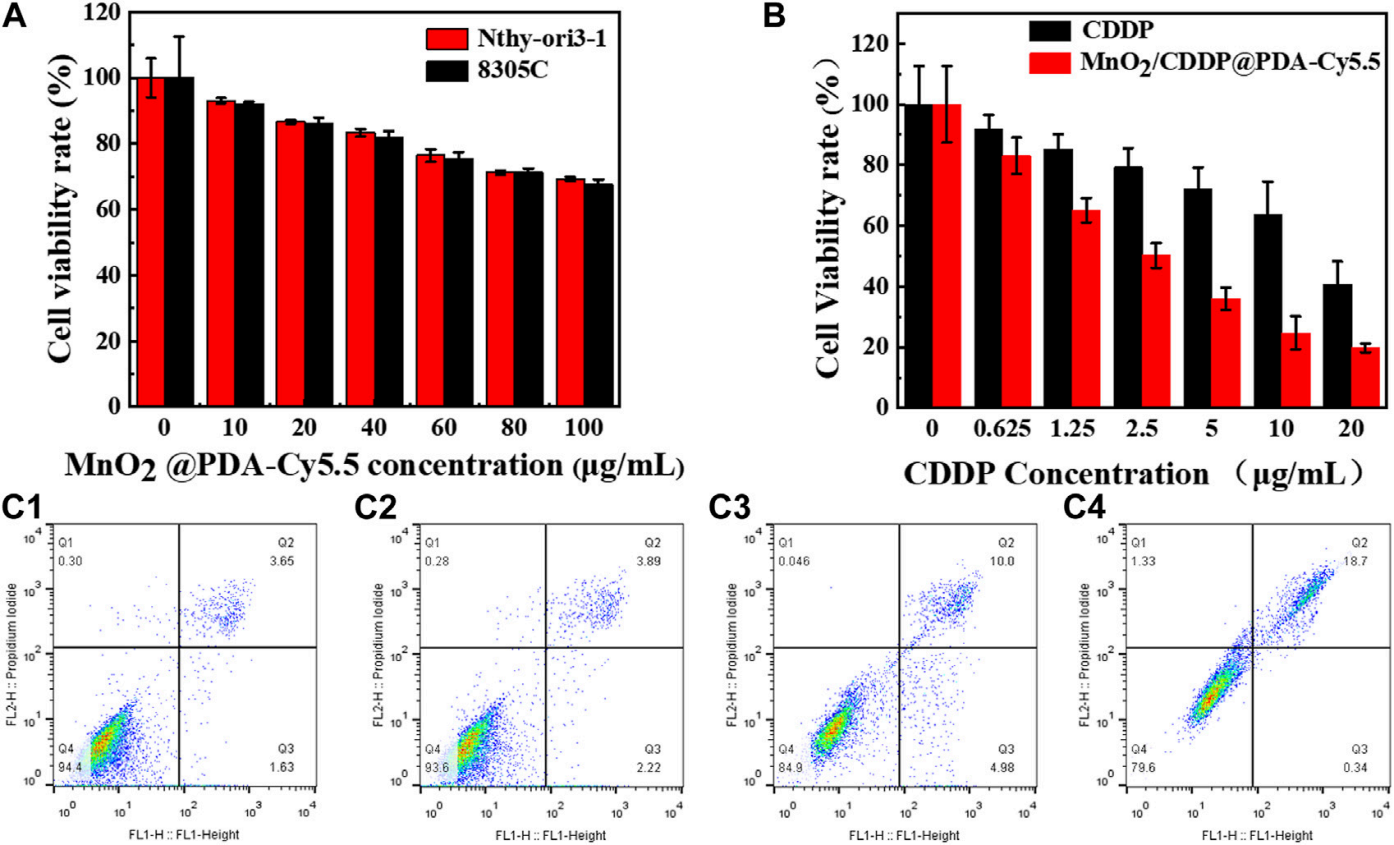
**(A)** Relative viability of 8305C and Nthy-ori3-1 cells after incubation with different concentrations of MnO_2_@PDA-Cy5.5 for 24 h. **(B)** Relative viability of 8305C cells after incubation with free CDDP and MnO_2_/CDDP@PDA-Cy5.5 at different CDDP concentrations. **(C**_**1**_ **–C**_**4**_ **)** The apoptosis rates of 8305C cells co-incubation with PBS, MnO_2_, free CDDP and MnO_2_/CDDP@PDA-Cy5.5 NPs for flow cytometry apoptosis data.

As depicted in Figure 6A, after 8305C cells and Nthy-ori3-1 cells were administrated with different concentrations of MnO_2_@PDA-Cy5.5 for 24 h, the survival rate of the cells reached nearly 70% even at the concentration of 100 μg/mL. As revealed by the above result, MnO_2_@PDA-Cy5.5 exhibited high biocompatibility with 8305C cancer cells and Nthy-ori3-1 normal, and it can serve as a drug carrier for in-depth research.

Subsequently, 8305C cells were adopted to evaluate the therapeutic effect of MnO_2_/CDDP@PDA-Cy5.5. As depicted in Figure 6B, with the increase of CDDP concentration, cell mortality in the MnO_2_/CDDP@PDA-Cy5.5 group was notably higher than that in the free CDDP group. Furthermore, at the identical concentration of CDDP, the mortality rate in the MnO_2_/CDDP@PDA-Cy5.5 group was significantly higher than that in the CDDP group, suggesting that MnO_2_/CDDP@PDA-Cy5.5 has a better ability to inhibit the growth of tumor cells. This can be attributed to the higher amount of CDDP that the drug delivery system is able to internalize in the cell.

### 3.7 Apoptosis experimental results

The results of flow cytometry are shown in Figure 6(C_1_-C_4_). The apoptosis rates of 8305C cells administrated with PBS, MnO_2_, CDDP and MnO_2_/CDDP@PDA-Cy5.5 were 5.28%, 6.11%, 14.98% and 19.04%, respectively. Compared with free CDDP, MnO_2_/CDDP@PDA-Cy5.5 induced more apoptosis in 8305C cells. This can be attributed to the EPR effect of nanoparticles improved the targeting of nanoparticles and effectively enhanced the apoptosis of 8305C cells.

### 3.8 *In vivo* NIR fluorescence imaging results

**FIGURE 7:**
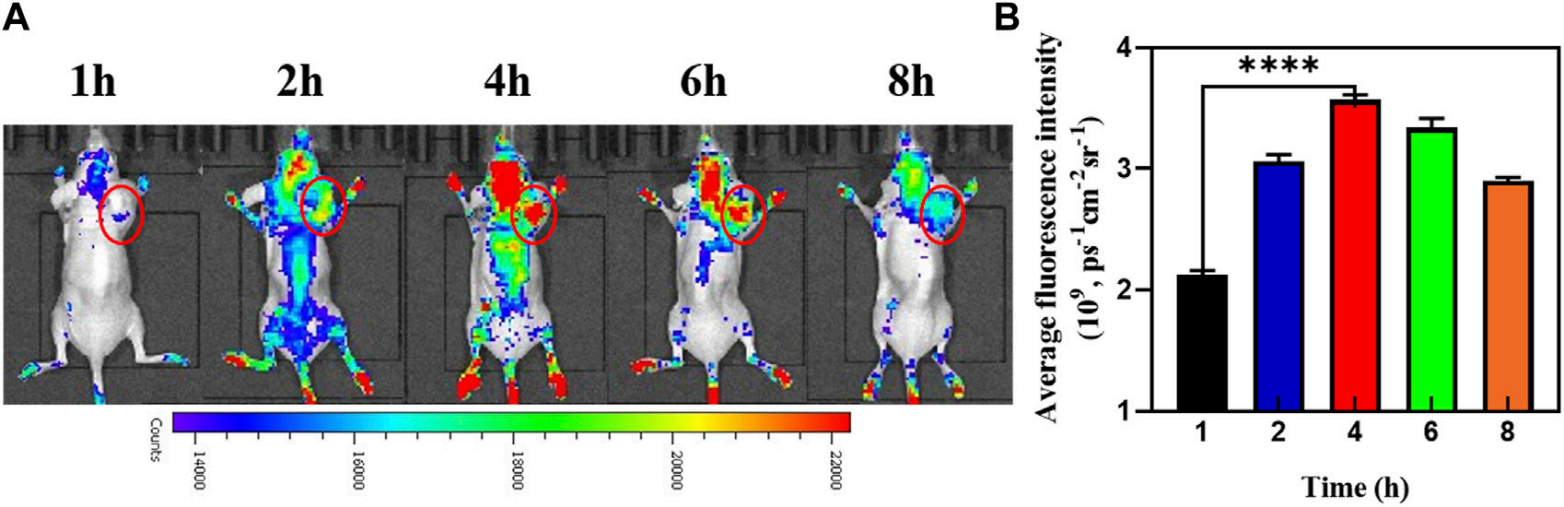
**(A)** *In vivo* fluorescence images of the mice at different times post-injection of MnO_2_/CDDP@PDA-Cy5.5 NPs. **(B)** Average fluorescence intensity of tumor areas at different time intervals based on *in vivo* fluorescence images shown in **(A)**.

As depicted in Figures 7A, B, whole-body fluorescence imaging showed widespread Cy5.5 fluorescence in the mouse body 1 h after an intravenous injection of MnO_2_/CDDP@PDA-Cy5.5. With the gradual accumulation and penetration of MnO_2_/CDDP@PDA-Cy5.5, the fluorescence signal was detected only in the tumor area and reached its maximum intensity 4 h after injection, suggesting effective tumor accumulation by MnO_2_/CDDP@PDA-Cy5.5 NPs through EPR effect. Notably, fluorescence at tumor sites was still evident 8 h after injection, arising from the prolonged blood circulation of NPs and their remarkable ability to target the tumor.

### 3.9 *In vivo* magnetic resonance imaging results

**FIGURE 8:**
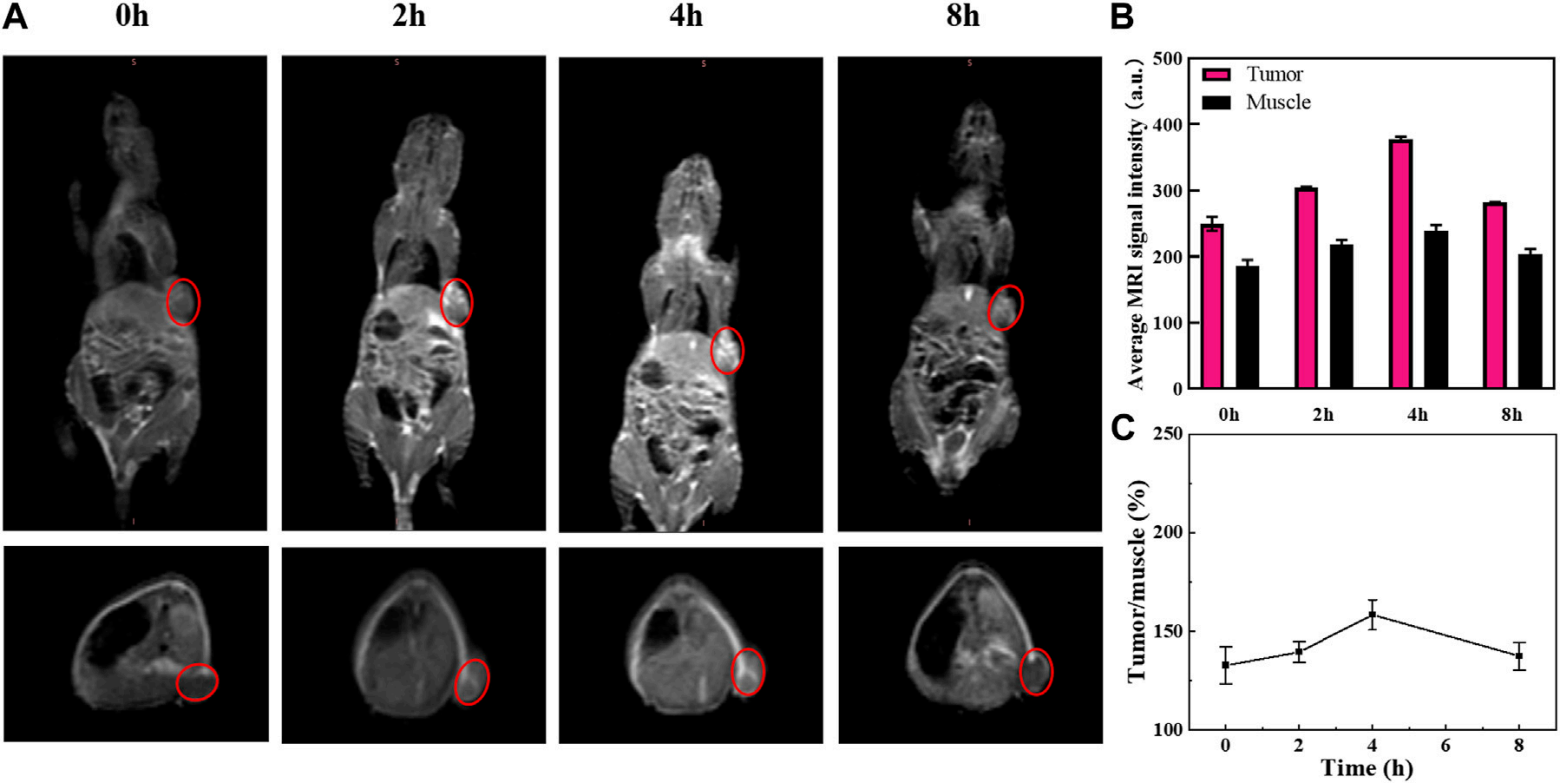
**(A)** T_1_-weighted MR images of 8305C tumor-bearing mice before and after intratumoral injection of MnO_2_/CDDP@PDA-Cy5.5. **(B)** Average MRI signal intensity of tumor and muscle at different time points. **(C)** Ratio of tumor to muscle T_1_ signal intensity based on Figure 8B.

The *in vivo* imaging of MnO_2_/CDDP@PDA-Cy5.5 is shown in Figure 8A. Following the injection of nanoparticles, the T_1_ imaging signal in the tumor steadily grew over time, which was peaked at 4 h. This is also confirmed by comparing the intensity of T_1_ signal corresponding to the tumor site and muscle Figures 8B, C. Accordingly, MnO_2_/CDDP@PDA-Cy5.5 could be employed as an acid-responsive T_1_ contrast agent.

### 3.10 *In vivo* antitumor effect

**FIGURE 9:**
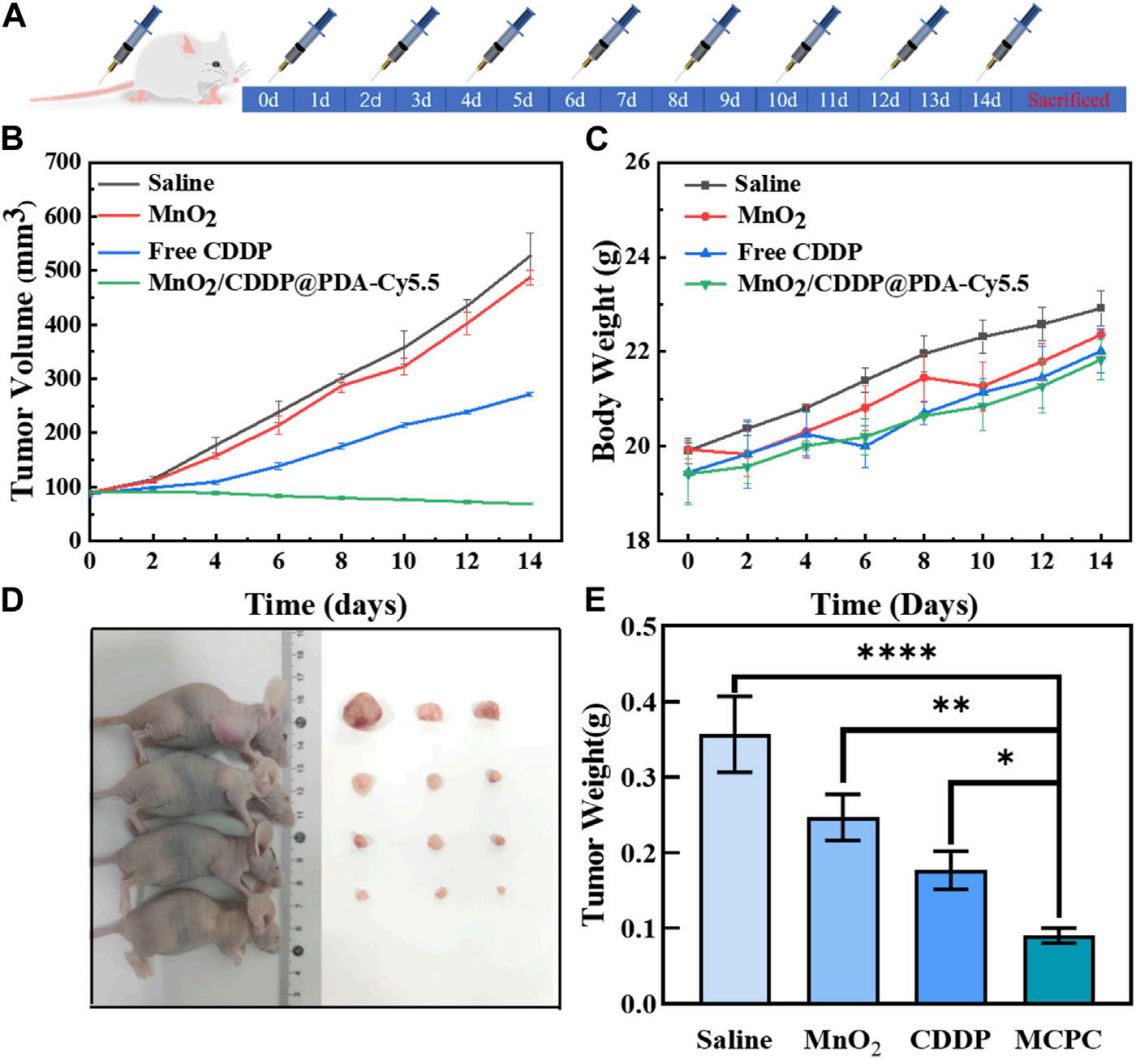
**(A)** Schematic diagram of treatment with PBS, MnO_2_, free CDDP and MnO_2_/CDDP@PDA-Cy5.5 in BAlB/c nude mouse tumor model. **(B)** Growth trend in tumor volume, **(C)** Body weight of nude mice in different treatment groups, **(D)** Tumor treatment renderings, and **(E)** final mean tumor weight after 14 days of treatment.

As depicted in Figure 9A, tumor bearing nude mice were divided into groups for 14 days of treatment. Figure 9B showed that no significant inhibitory effect was observed on tumor volume in nude mice in the PBS and MnO_2_ groups, but after 14 days of treatment, tumor growth rate in the free CDDP group was inhibited to a certain extent. In addition, tumor volume decreased in MnO_2_/CDDP@PDA-Cy5.5 nude mice. Meanwhile, as depicted in Figure 9C, there was no significant difference in body weight among the groups. The mice were killed at the conclusion of the trial, and the tumors were removed, photographed, and then weighed. As depicted in Figure 9D, the MnO_2_/CDDP@PDA-Cy5.5 group exerted the optimal therapeutic effect. Figure 9E illustrates the final tumor weight. The mean tumor weight of the PBS group, the MnO_2_ group, the free CDDP group, and the MnO_2_/CDDP@PDA-Cy5.5 group reached 0.367 g, 0.227 g, 0.132 g, and 0.100 g, respectively, consistent with the growth trend of tumor volume. As revealed by the above-mentioned results, MnO_2_/CDDP@PDA-Cy5.5 can exert a notable therapeutic effect while outperforming free CDDP.

### 3.11 Biodistribution and safety evaluation

As depicted in Supplementary Figure S9, the kidneys of the free CDDP group had tubular collapse. The renal tubules in the MnO_2_/CDDP@PDA-Cy5.5 group were organized in comparison to the free CDDP group, and no pathological alterations or necrosis were reported in the kidneys, suggesting that MnO_2_/CDDP@PDA-Cy5.5 mitigated the renal toxicity of CDDP. There were no significant differences in all other major organs, including the heart, liver, spleen and lungs, in the MnO_2_ or MnO_2_/CDDP@PDA-Cy5.5 groups compared with the saline group.

**FIGURE 10:**
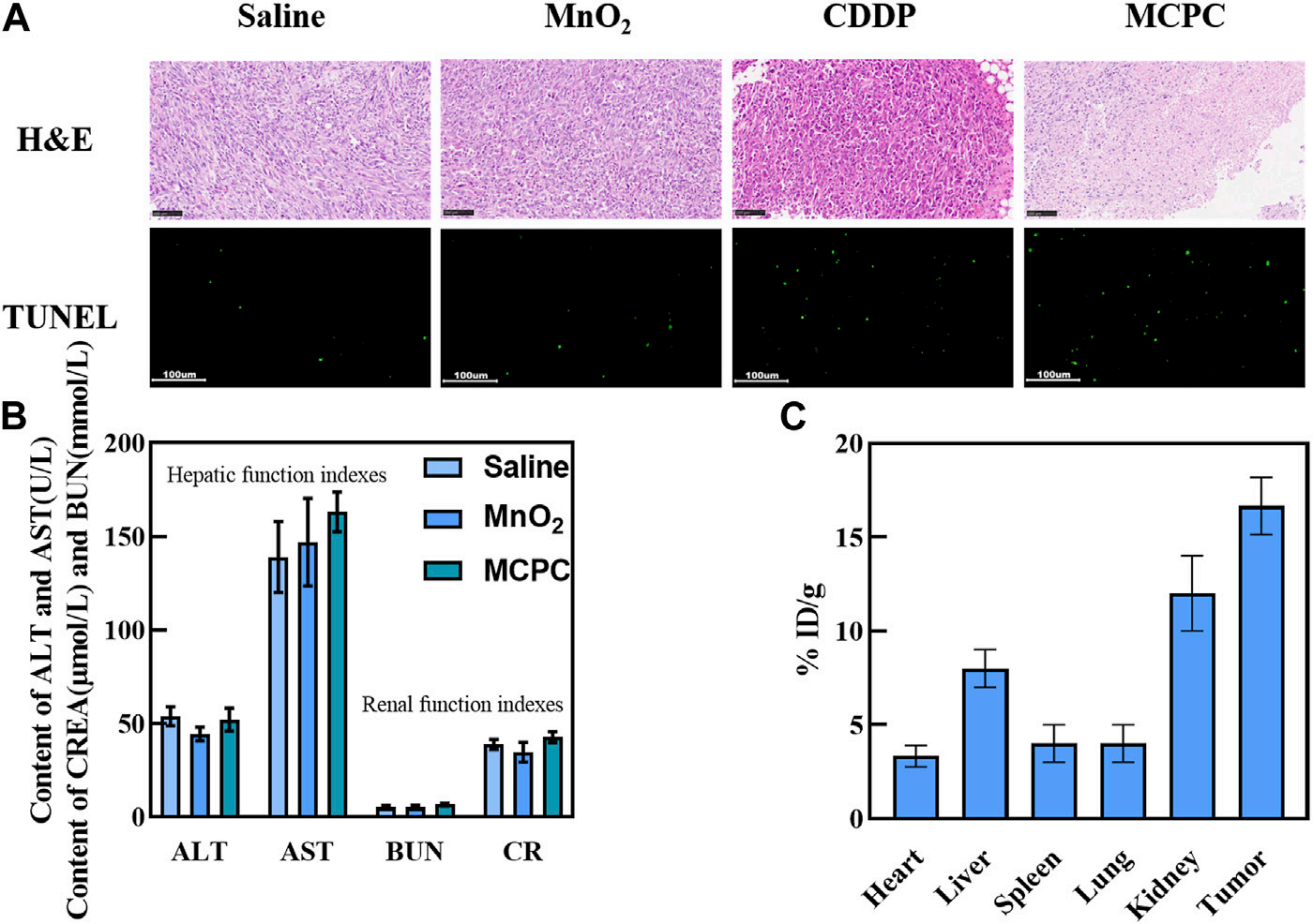
**(A)** H&E staining and TUNEL assay performed on the tumor slices after the experiment. **(B)** Blood biochemical analyses of the mice administrated with Saline, MnO_2_ and MnO_2_/CDDP@PDA-Cy5.5 NPs. **(C)** The distribution of the NPs at 24 h post-injection, quantified in terms of Pt concentration.

Figure 10A presents the representative tumor H&E staining samples, suggesting that the MnO_2_/CDDP@PDA-Cy5.5 group had tumor cell necrosis and shrinkage compared with the rest groups. Thus, the significantly improved therapeutic efficacy was shown for tumor-bearing mices administered MnO_2_/CDDP@PDA-Cy5.5 in comparison to those administered with CDDP at the identical CDDP doses. Based on *in situ* TUNEL, the apoptosis levels of tumor tissues were investigated in depth, and little green coloration was observed in the tumors of nude mice administrated with PBS and MnO_2_, suggesting that no cell apoptosis was identified (Figure 10A). In contrast, tumors of nude mice administrated with free CDDP displayed an increase in the amount of green cells, and the highest level of apoptosis was observed in tumors of nude mice administrated with MnO_2_/CDDP@PDA-Cy5.5 NPs. Thus, compared with free CDDP, MnO_2_/CDDP@PDA-Cy5.5 nanoparticles are more effective in inducing apoptosis.

As depicted in Figure 10B, compared with the control group, the indexes of liver function (ALT and AST) and renal function (CREA and BUN) in the experimental group are very similar to those in the control group. Moreover, referring to the indexes of liver and kidney in the literature, it was found that MnO_2_/CDDP@PDA-Cy5.5 NPs did not induce obvious liver and kidney dysfunction (Rong et al., 2021). In a word, the above-mentioned results show that MnO_2_/CDDP @ PDA-Cy5.5 NPS has good biocompatibility, and can provide passive drug delivery with low risk of off-target toxicity.

The biodistribution results indicated that much more Pt^2+^ existed in the tumor than in the other organs, suggesting that MnO_2_/CDDP@PDA-Cy5.5 NPs exhibited significant tumor accumulation (Figure 10C), probably due to the EPR effect. Moreover, high amounts of Pt^2+^ were identified in the kidneys of the above-mentioned mice, suggesting that Pt^2+^ from MnO_2_/CDDP@PDA-Cy5.5 NPs was quickly cleared by the kidneys.

## 4 Conclusion

The MnO_2_ nanospheres prepared using the template method were spherical with hollow structure under the transmission electron microscope, and the average particle size reached nearly 70 nm. The stability of MnO_2_/CDDP@PDA-Cy5.5 NPs was notably enhanced after polydopamine modification, the particle size of the prepared MnO_2_/CDDP@PDA-Cy5.5 NPs was 90 nm, and the Zeta potential was 28.9 ± 3.90 mV. The results of drug release experiments showed that MnO_2_/CDDP @ PDA-Cy5.5 released CDDP rapidly under acidic and redox conditions, and the cumulative release reached its maximum at 8 h. As indicated by the magnetic resonance imaging results, the prepared drug delivery system was pH and redox responsive, and the degraded Mn^2+^ could be employed for MRI imaging. *In vitro* cytological research on MnO_2_/CDDP@PDA-Cy5.5 nanoparticles suggested that the carrier was biocompatible with 8305C cells and MnO_2_/CDDP@PDA-Cy5.5 could effectively kill cancer cells *in vitro*. Besides, a high level of uptake of MnO_2_/CDDP@PDA-Cy5.5 NPs by cancer cells was identified through confocal microscopy. As revealed by the *in vivo* zoological research on MnO_2_/CDDP@PDA-Cy5.5 NPs, the drug delivery system can stably deliver drugs to the tumor site, thus exerting significant inhibitory effect on the growth of the tumor. Furthermore, the drug delivery system can be employed for magnetic resonance imaging after being degraded to Mn^2+^ at the tumor site.

The overall results suggested that the drug delivery system has pH and redox responsiveness, and can deliver drugs to tumor sites stably, and the Mn^2+^ produced by degradation exhibited the capability of magnetic resonance imaging, such that the dual functions of tumor diagnosis and treatment can be achieved, and a certain theoretical and practical basis can be laid for the research of new preparations for tumor targeted diagnosis and treatment.

## Data Availability

The original contributions presented in the study are included in the article/Supplementary Material, further inquiries can be directed to the corresponding authors.
