# Supplementary material for: Redox/pH-responsive hollow manganese dioxide nanoparticles for thyroid cancer treatment
Source: Front Chem. 2023 Sep 15;11:1249472. doi: 10.3389/fchem.2023.1249472 (PMC10540626; doi:10.3389/fchem.2023.1249472)
Supplement: Supplementary file 1 [file DataSheet1.PDF]

Supplementary Material

pH-responsive hollow manganese dioxide is used in the treatment of thyroid cancer

Jinren Liu, Changzhi Guo, Chunxiang Li, Qiushi Jia, Zhengrong Xie, Ziyue Wang, Hongda Tian, Zhongyuan Li* and Liguo Hao*

*** Correspondence:** Corresponding Author: m15946506063@163.com and haoliguo@qmu.edu.cn

1 Supplementary Figures and Tables

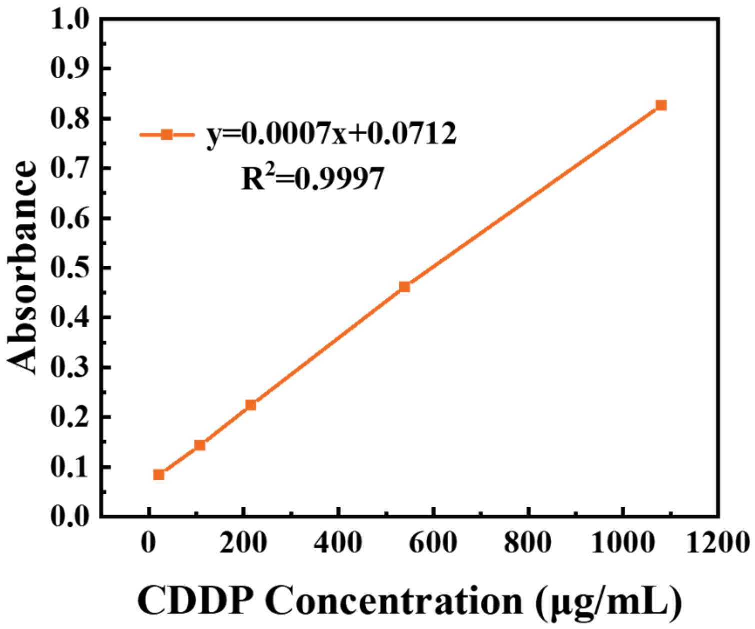

Figure S1 Standard curve of CDDP.

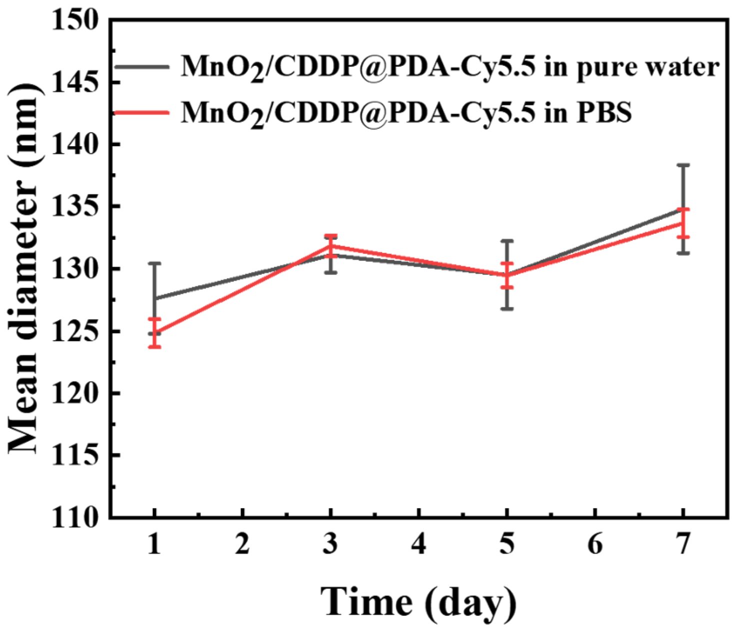

Figure S2 Particle size change of the MnO_2 /CDDP@PDA-Cy5.5 NPs in pure water and PBS for 7 days.

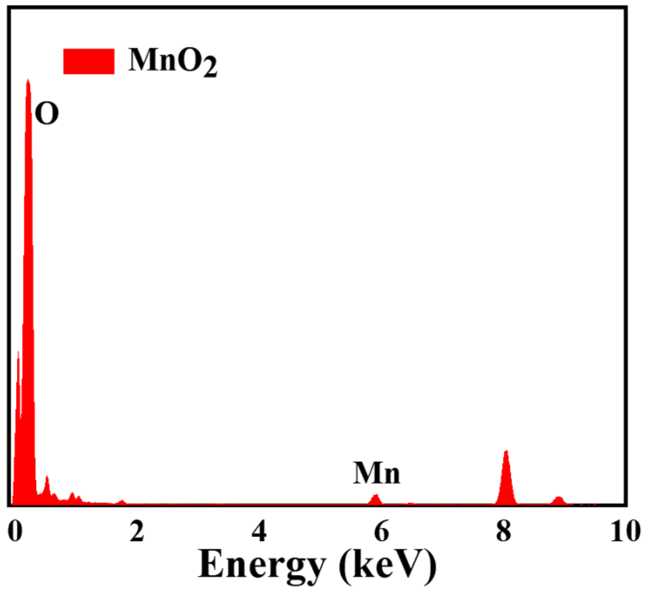

Figure S3 EDS pattern of MnO_2 NPs.

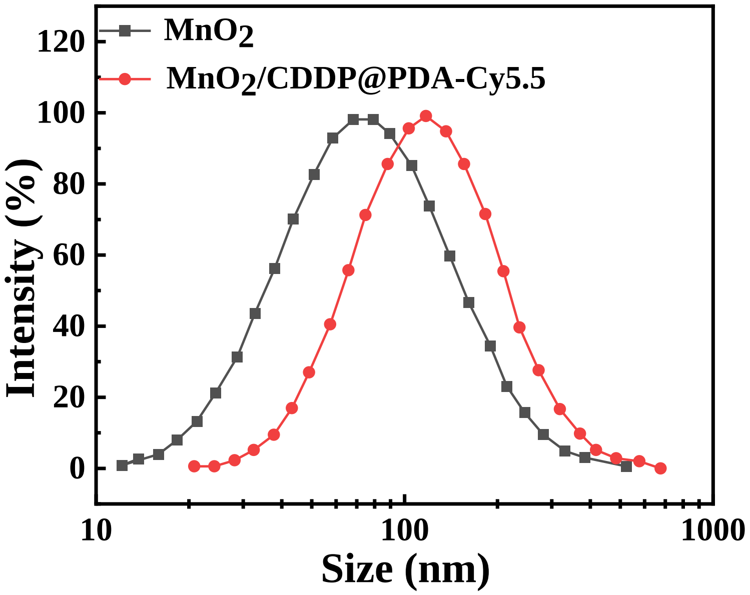

Figure S4 Hydrated particle size of MnO_2 and MnO_2/CDDP@PDA-Cy5.5 NPs

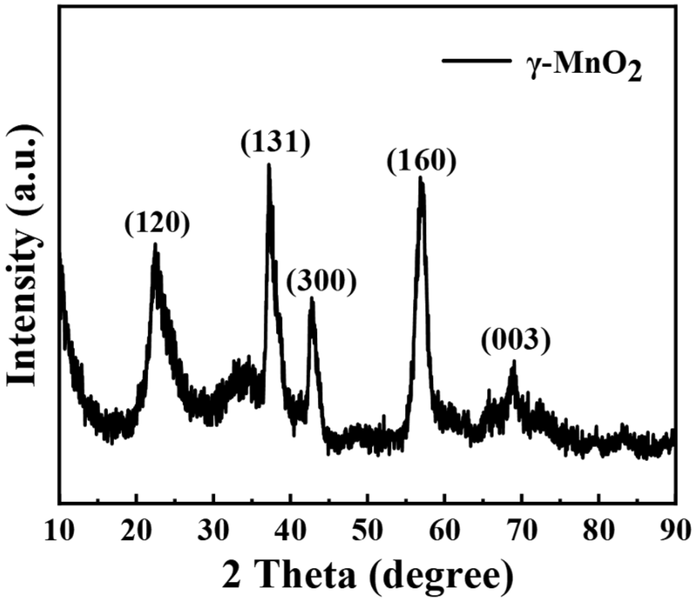

Figure S5 XRD pattern of MnO_2

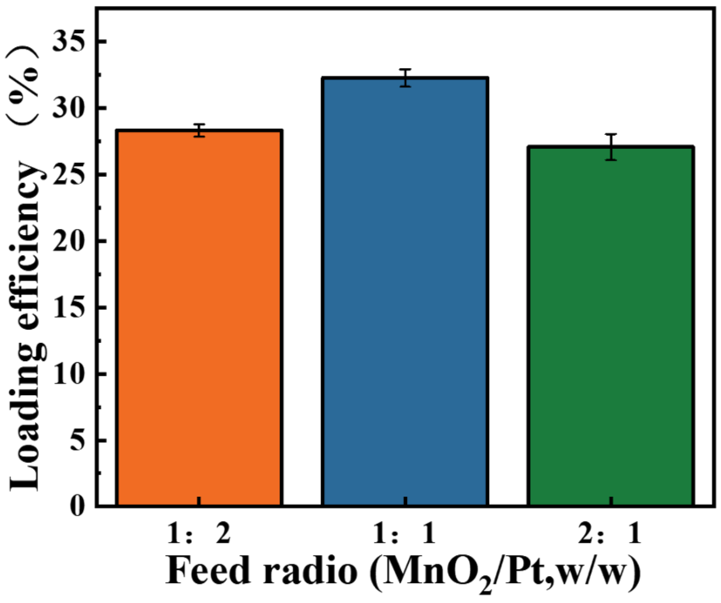

Figure S6 loading efficiency of CDDP at different feed ratios.

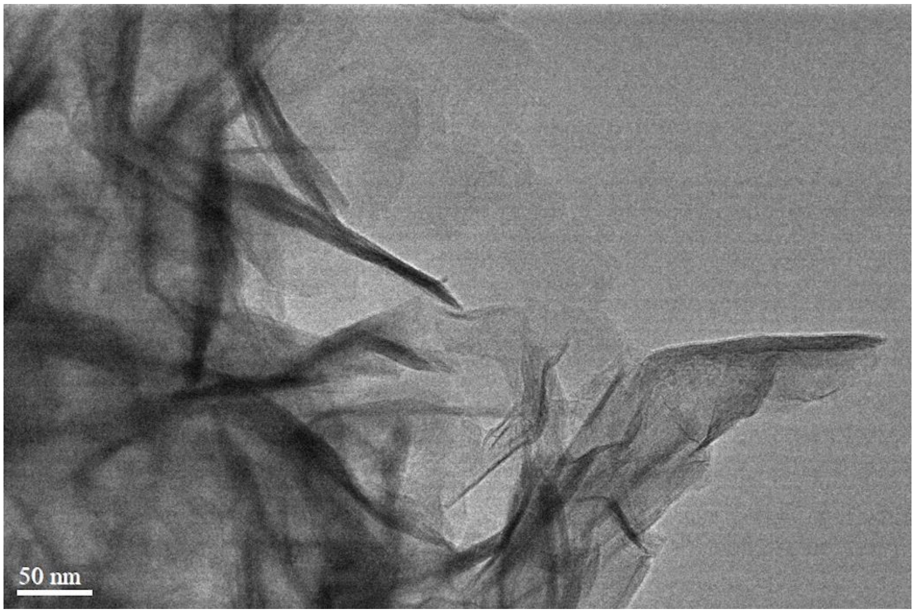

Figure S7 TEM of MnO_2 /CDDP@PDA-Cy5.5 degradation in acidic solution (pH 5.5).

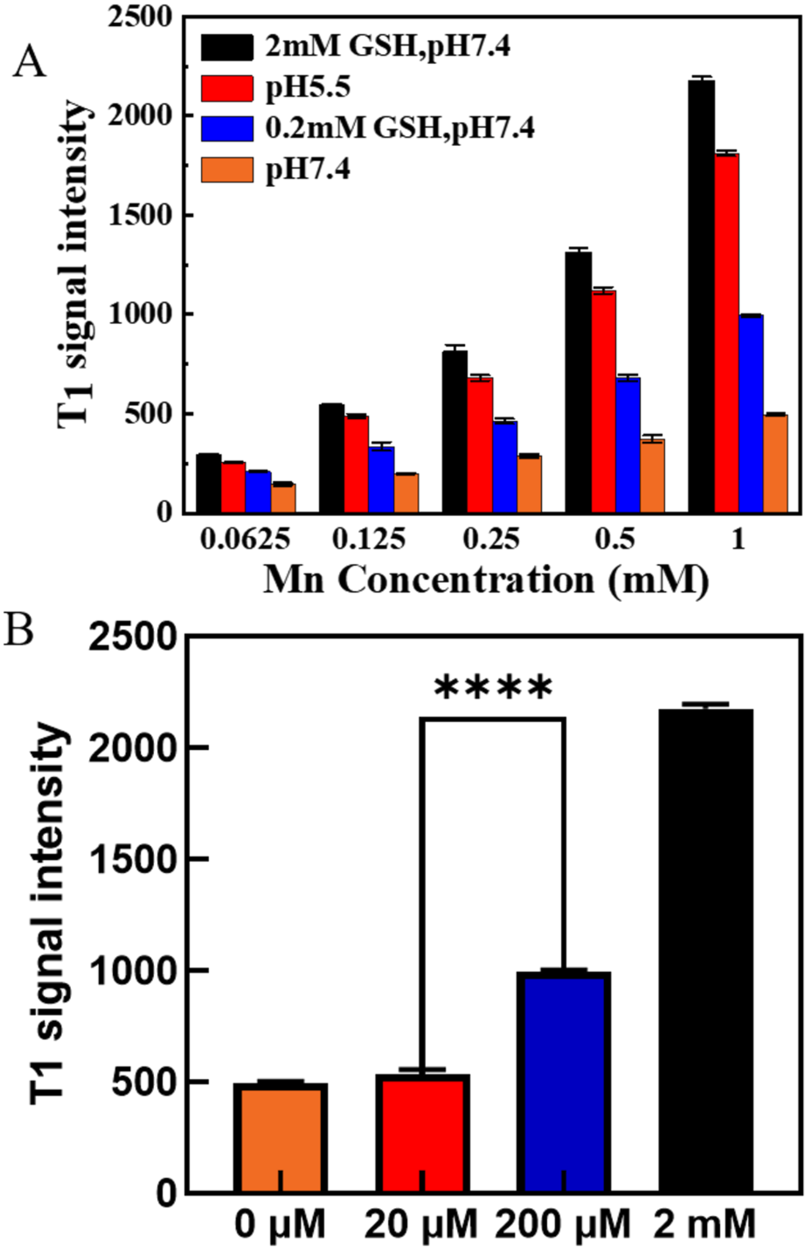

Figure S8 (A) MR signals of MnO_2/CDDP@PDA-Cy5.5 NPs under different conditions. (B) MR signals of MnO_2/CDDP@PDA-Cy5.5 NPs in the presence of different concentrations of GSH (0-2 mM) at pH=7.4, at 3 T.

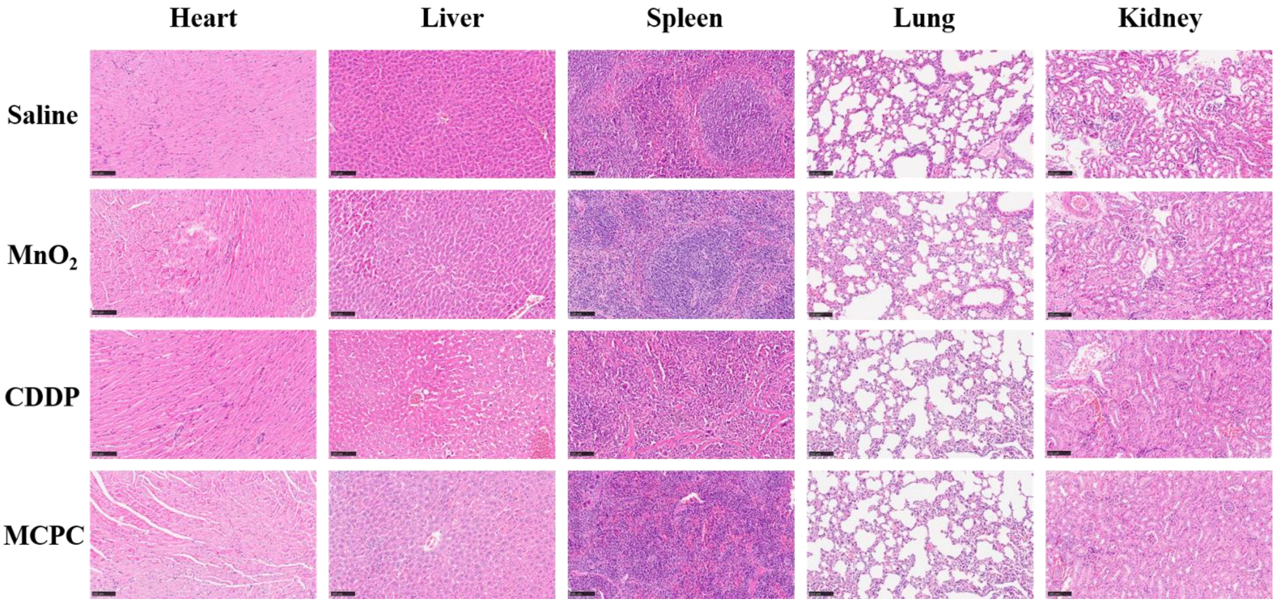

Figure S9 H&E staining of heart, liver, spleen, lung, kidney and tumor sections collected from mice in each group after the process (scale: 100μm).
